# The Sparrow and the Cat

**Published:** 1884-05

**Authors:** 


### THE SPARROW AND THE CAT.

THEY say: A cat once caught a sparrow and was about to devour it when the sparrow spoke up and said, "No gentleman eats till he has first washed his face." The cat was struck with this sage remark, sat the sparrow down and began to wash his face with his paw. The sparrow flew away. This enraged pussy, and he swore —"As long as I live I will eat first, and wash my face afterwards." And cats have done so ever since.

